# Subcellular Fractionation and Localization Studies Reveal a Direct Interaction of the Fragile X Mental Retardation Protein (FMRP) with Nucleolin

**DOI:** 10.1371/journal.pone.0091465

**Published:** 2014-03-21

**Authors:** Mohamed S. Taha, Kazem Nouri, Lech G. Milroy, Jens M. Moll, Christian Herrmann, Luc Brunsveld, Roland P. Piekorz, Mohammad R. Ahmadian

**Affiliations:** 1 Institute of Biochemistry and Molecular Biology II, Medical Faculty of the Heinrich-Heine-University, Düsseldorf, Germany; 2 Laboratory of Chemical Biology and Institute of Complex Molecular Systems, Department of Biomedical Engineering, Technische Universiteit Eindhoven, Eindhoven, the Netherlands; 3 Department of Physical Chemistry I, Ruhr University Bochum, Bochum, Germany; CNRS UMR7275, France

## Abstract

Fragile X mental Retardation Protein (FMRP) is a well-known regulator of local translation of its mRNA targets in neurons. However, despite its ubiquitous expression, the role of FMRP remains ill-defined in other cell types. In this study we investigated the subcellular distribution of FMRP and its protein complexes in HeLa cells using confocal imaging as well as detergent-free fractionation and size exclusion protocols. We found FMRP localized exclusively to solid compartments, including cytosolic heavy and light membranes, mitochondria, nuclear membrane and nucleoli. Interestingly, FMRP was associated with nucleolin in both a high molecular weight ribosomal and translation-associated complex (≥6 MDa) in the cytosol, and a low molecular weight complex (∼200 kDa) in the nucleoli. Consistently, we identified two functional nucleolar localization signals (NoLSs) in FMRP that are responsible for a strong nucleolar colocalization of the C-terminus of FMRP with nucleolin, and a direct interaction of the N-terminus of FMRP with the arginine-glycine-glycine (RGG) domain of nucleolin. Taken together, we propose a novel mechanism by which a transient nucleolar localization of FMRP underlies a strong nucleocytoplasmic translocation, most likely in a complex with nucleolin and possibly ribosomes, in order to regulate translation of its target mRNAs.

## Introduction

Fragile X syndrome (FXS) is one of the most common forms of inherited mental retardation, which is associated with various behavioral and physiological abnormalities, including social withdrawal, anxiety, intellectual disability, epilepsy and autism [1], [2], [3]. FXS is caused by the absence of the fragile X mental retardation protein (FMRP) [4], [5], [6], which belongs to the RNA-binding, fragile X related protein (FXRP) family that includes also the fragile X related proteins 1 and 2 (FXR1P and FXR2P) [7], [8].

FMRP is ubiquitously expressed with higher abundance in the brain and testis [7], [9]. Studies by a number of laboratories have shown that FMRP is a regulator of protein translation and associates with the translation machinery [4], [10]. FMRP is associated with messenger ribonucleoprotein (mRNP) particles and large polyribosomal complexes in the cytoplasm of various cell types [11], [12], [13], [14]. FMRP consists of an N-terminal dimerization domain, a central region containing two K homology (KH1 and KH2) domains and a C-terminus encompassing the arginine-glycine-glycine (RGG) region [15], [16]. The N-terminal and central regions of FMRP are highly conserved among the FXRPs, while the C-terminal shows significant variability [7]. FMRP is known to play roles in nucleocytoplasmic shuttling of mRNA by a non-canonical nuclear localization signal (NLS) and a nuclear export signal (NES) [17], [18], [19], [20]. Different mechanisms for the nuclear export of FMRP have been suggested involving CRM1/exportin1 [20] and/or the nuclear export factor family proteins [17]. In addition, FXR1P and FXR2P have been reported to contain a nucleolar localization signal (NoLS) at their C-termini, which is not reported yet in FMRP [7] although its nucleolar localization has been described previously [21].

Several FMRP interacting proteins have been identified so far. FXR1P and FXR2P are structurally and functionally related to FMRP. They additionally harbor a functional nucleolar targeting signal [7]. The cytoplasmic interacting FMR1 protein (CYFIP; also known as p140 and PIR121, respectively), a binding partner of FMRP [22], acts as a downstream effector of Rac1 thereby linking Rac1 to actin dynamics and lamellipodia formation. Activated Rac1 binds CYFIP and sequesters it from its complex with FMRP, which in turn is then released to regulate protein translation [23]. Moreover, nuclear FMRP interacting protein 1 (NUFIP1) has been identified as a nuclear RNA binding protein [24]. Other molecules described in FMRP protein complexes include the RNA-induced silencing complex (RISC), argonaute 2 (AGO2), Dicer, the 82-kDa FMRP interacting protein (82-FIP), as well eukaryotic initiation factor 5 (eIF5) and nucleolin [25], [26], [27].

When six different cell lines were analyzed, i.e. Cos-7, HEK 293, HeLa, MDCK II, MEF, and NIH3T3, we found the strongest FMRP expression in HeLa cells. Thus, we investigated in this study the subcellular localization and interaction of FMRP with its binding partners in HeLa cells using an anti-FMRP antibody (ab17722) that has been successfully analyzed in FMR1 knockout mice [3]. Our novel findings reveal that FMRP localizes predominantly in different cellular compartments, including mitochondria and nucleoli. Interestingly, FMRP was found to be associated with nucleolin in two distinct protein complexes, a cytosolic high molecular weight translation-associated complex and a nucleolar, low molecular weight complex. Using purified proteins we show that the N-terminus of FMRP undergoes a direct protein-protein interaction with the C-terminal RGG domain of nucleolin. We further demonstrate that the nucleolar localization of FMRP is specifically regulated by two functional NoLSs in its C-terminus.

## Results and Discussion

### FMRP localization at different subcellular compartments in HeLa cells

**Figure 1:**
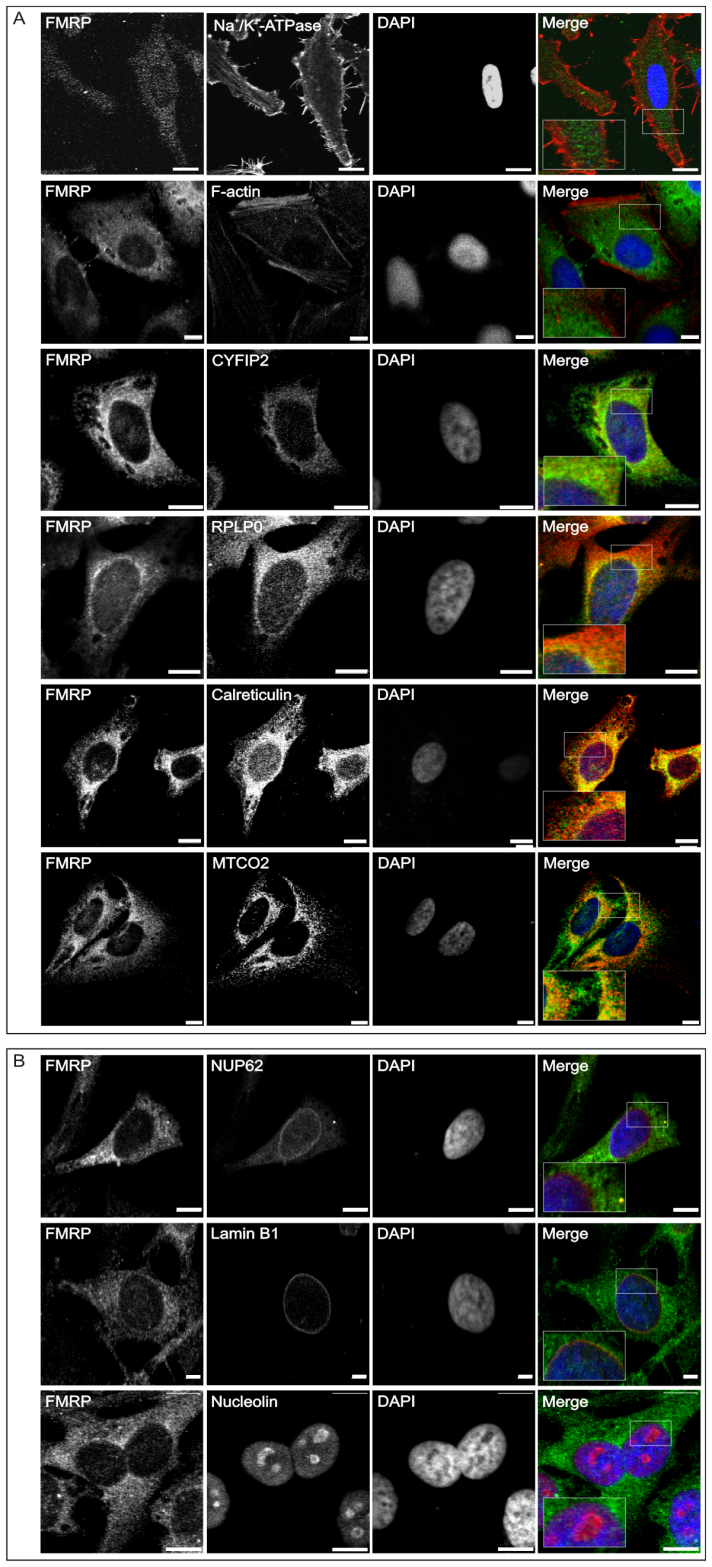
FMRP is localized at various intracellular sites in HeLa cells. Confocal laser scanning microscopy (cLSM) images of HeLa cells depicting endogenous FMRP (green channel) costained with various cytosolic (A) and nuclear (B) markers (red channel), including antibodies against CYFIP2, RPLP0 (ribosomal proteins), nucleolin (nucleolar marker), MTCO2 (mitochondrial protein), NUP62 (nucleoporins), lamin B1 (nuclear intermediate filament proteins), and calreticulin (endoplasmic reticulum marker). Detection of Na^+^/K^+^-ATPase and phalloidin staining were used to detect the cellular membrane and F-actin, respectively. DNA was stained by using DAPI (blue channel). Boxed areas in the merged panels depict enlarged areas of interest. Scale bar: 10 μm.

In a first step we employed confocal laser scanning microscopy (cLSM) to analyze the intracellular distribution of endogenous FMRP in HeLa cells. Cells were co-stained with antibodies specific for several endogenous markers, i.e. cellular organelles and compartments. As indicated in Figure 1, FMRP can be detected at almost every compartment of the cell. No colocalization could be found with the plasma membrane and filamentous actin (F-actin) when costained with antibodies against the transmembrane protein Na^+^/K^+^-ATPase and fluorescent phalloidin, respectively (Fig. 1A). FMRP considerably colocalized in perinuclear regions and at endomembranes with its binding partner CYFIP, the 60S acidic ribosomal protein p0 (RPLP0), the eukaryotic initiation factor 5 (eIF5) [26], and calreticulin (a marker of the endoplasmic reticulum). Of note, the mitochondrially encoded cytochrome c oxidase subunit II (MTCO2; also known as COX-2) showed a clear colocalization with FMRP suggesting that FMRP may be associated with the mitochondrial (see below).

Only a very weak colocalization could be detected under these conditions when employing markers for the nuclear membrane (NUP62) as well as antibodies specific for lamin B1 and surprisingly, also nucleolin (Fig. 1B). Interestingly, nucleolin has been previously reported to be part of FMRP protein complexes linked to translational inhibition [26]. Although FMRP was not clearly detectable in the nucleus under these conditions, it is important to note that FMRP contains both NLS and NES sequences [15], with the latter playing a putative role in nucleocytoplasmic shuttling of ribosomes probably in association with nucleolin [26].

### Subfractionation and biochemical characterization of intracellular FMRP localization

**Figure 2:**
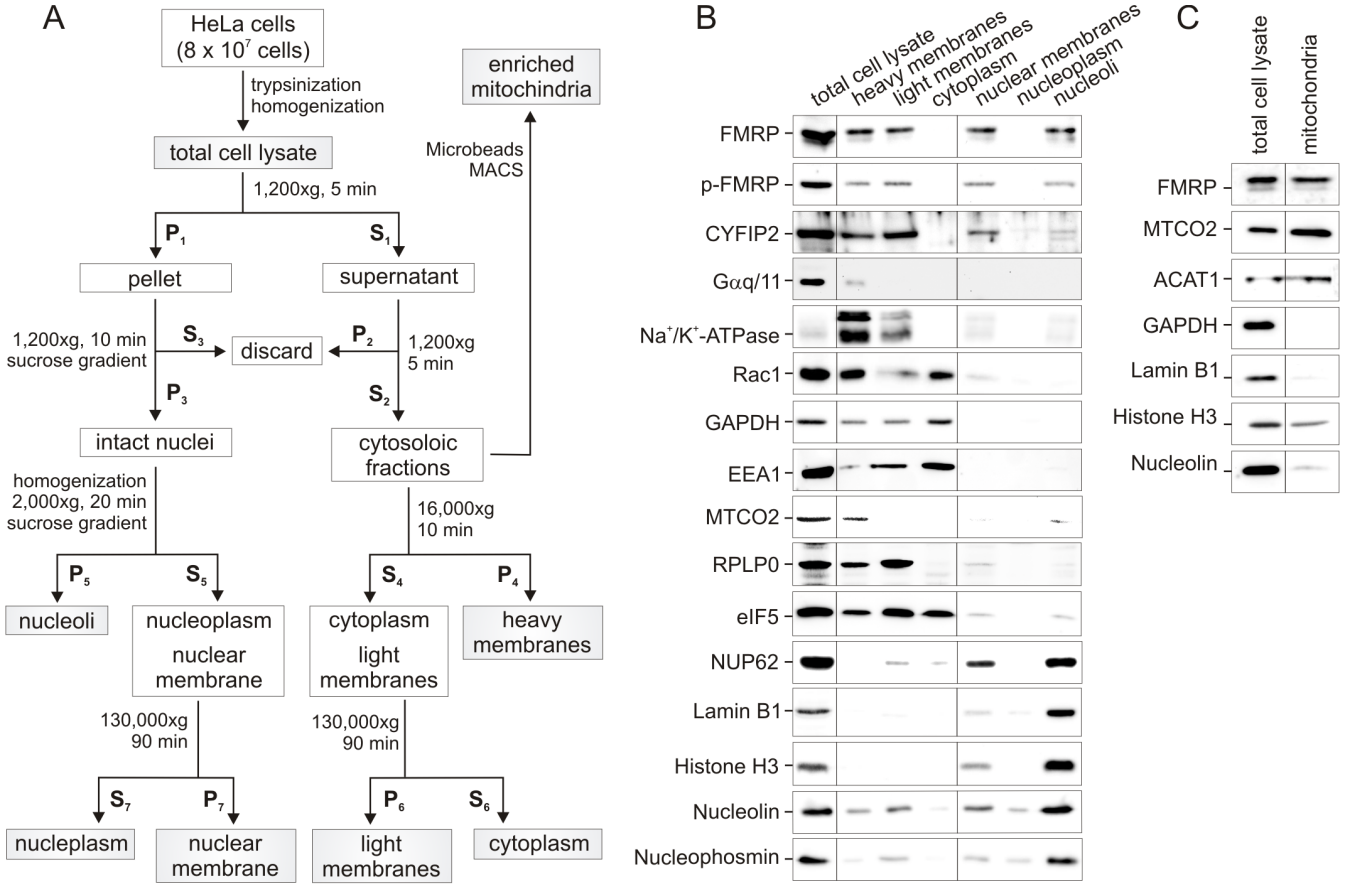
FMRP shows a diverse subcellular distribution pattern in HeLa cells as revealed by subcellular fractionation analysis. (A) Experimental cell fractionation procedure employing several differential centrifugation steps. Cells were fractionated into six distinct fractions, including heavy membrane (plasma membrane and rough endoplasmic reticulum), light membrane (polysomes, golgi apparatus, smooth endoplasmic reticulum), cytoplasm (cytoplasm and lysosomes), enriched nuclear membrane (containing rough endoplasmic reticulum), nucleoplasm, and nucleoli. S, supernatant; P, pellet. (B) FMRP is largely absent in the cytoplasm and nucleoplasm and predominantly localizes to solid compartments. The protein concentrations were normalized in all fractions with exception of the nucleoplasm due its low protein content as compared to the other fractions. In each lane, 5 μg proteins were loaded except for the nucleoplasm, where one μg was used. In addition to FMRP and its binding partner CYFIP, the fractions were analyzed by using different subcellular marker, including Gα_q/11_, Na^+^/K^+^-ATPase and Rac1 (plasma membrane), EEA2 (endosomes), GAPDH (cytoplasm), eIF5 and RPLP0 (ribosomes and rough ER). Nuclear markers included histone H3 and lamin B1. Nucleolin was used as nucleolar marker. (C) Detection of FMRP in mitochondria. The presence of FMRP in isolated mitochondrial fraction was analyzed by SDS-PAGE and immunoblotting, using antibodies against FMRP, two mitochondrial proteins MTCO2 and ACAT1, the cytosolic GAPDH as well as the nuclear proteins lamin B1, histone H3 and nucleolin. Equal protein amounts of the mitochondrial fraction and the total cell lysate were used.

Next, the cellular compartmentalization of FMRP and its interacting partners in HeLa cells was investigated in greater detail by establishing a detergent-free differential centrifugation protocol through sucrose cushions as described in Material and Methods. Thereby, we obtained six distinct fractions, including heavy membrane fraction (plasma membrane, mitochondria and rough endoplasmic reticulum or rER), light membrane fraction (smooth endoplasmic reticulum or sER, and free polysomes), cytoplasm fraction including lysosomes, nuclear membranes together with rER attached to the outer nuclear membrane, and lastly fractions containing the nucleoplasm and nucleoli (Fig. 2A). To evaluate the separation quality of the isolated sub-cellular fractions we used antibodies directed against specific marker proteins (Fig. 2B). The transmembrane protein Na^+^/K^+^-ATPase that is also expressed in HeLa cells, was consistently found in the heavy membrane and the light membrane fractions. Trace amounts of Na^+^/K^+^-ATPase were detected in the nuclear fractions, where it has been suggested to play a physiological role in Ca^2+^ homeostasis [28]. Another membrane marker found in the heavy membrane fraction, which undergoes palmitoylation and localizes to the plasma membrane [29], is α subunit of the G_q/11_ protein (Gα_q/11_). Glyceraldehyde-3-phosphate dehydrogenase (GAPDH) is largely present in the cytoplasmic fraction with comparable amounts also in heavy and light membranes. GAPDH has been reported to bind specifically to certain integral membrane proteins that are located in the plasma membrane, such Na^+^/K^+^-ATPase [30]. GAPDH is also associated with the GTPase Rab2 at the ER and Golgi apparatus [31]. The early endosome antigen 1 (EEA1) was used as endosomal marker to show that endosomes primarily exist in the cytoplasm and light membrane fractions [32]. NUP62 as a marker for the nuclear membrane, as well as lamin B1 and histone H3 as nuclear markers were employed and were also detected in the nucleolar fraction and the nuclear membrane as well as in the nucleoplasm in the case of lamin B1 [33]. Lastly, nucleolin and nucleophosmin (also called B23, NO38 or numatrin) were used as nucleolar markers. Both proteins were found not only in nuclear fractions but also in the light and heavy membranes. Interestingly, similar to nucleolin, also nucleophosmin has been implicated in the modulation of multiple cellular processes outside the nucleus [34].

Immunoblot analysis of all fractions using the above mentioned marker proteins revealed that FMRP and phospho-FMRP (Serine 499) exists predominantly in solid compartments, such as the cytosolic heavy and light membranes, the nuclear membrane, and also the nucleoli (Fig. 2B). FMRP appeared on the immunoblots in two major bands with molecular masses of 72 and 80 kDa, consistent with several previous studies [8], [9], [12], [26], [35], [36]. The fact that FMRP is part of a large mRNP complex (>600 kDa) [12] strongly indicates that the amounts of soluble FMRP must be very low. Consistent with this, no significant amounts of FMRP were detected in both the cytoplasm and in the nucleoplasm (Fig. 2B). The FMRP content in the respective fractions was calculated using the following approach: (i) The relative intensity of each protein band was determined by densitometric evaluation of FMRP immunoblot signal intensities; (ii) obtained intensities of each fraction were divided by the intensity obtained for FMRP in the total cell lysate and multiplied by the protein amounts used in every fraction; (iii) the obtained FMRP concentration in each fraction was divided by the total FMRP concentration and multiplied by 100. Accordingly, the FMRP content was 42.5% in the heavy membrane fraction, 22.5% in light membranes, 13.3% in the nuclear membrane fraction, and 21.7% in the nucleoli-containing fraction. The nucleolin content in these fractions, calculated in the same way, were 15.9%, 20.8%, 13.3% and 50.0%, respectively.

In addition to FMRP, we also analyzed the presence of proteins by immunoblot detection, which are either part of the translational machinery or known to modulate FMRP function in a RNA-dependent manner. Interestingly, the small GTPase Rac1 and its effector CYFIP revealed fractionation patterns, which were very similar to FMRP. CYFIP isoforms 1 and 2 were previously found in a complex with FMRP in HeLa cells [37], suggesting that FMRP may not discriminate between both highly conserved CYFIP isoforms. However, in contrast to CYFIP, Rac1 was found not only in the heavy and light membrane fractions, but also in the cytoplasm. Cytoplasmic Rac1 exists in complex with its regulator guanine nucleotide dissociation inhibitor GDlα [38]. Nuclear shuttling of Rac1 depends on its C-terminal polybasic region [39]. The nuclear import receptor karyopherin α2 has been shown to directly bind to and to translocate Rac1 into the nucleus [40]. It has been reported that GFP-CYFIP2 accumulates in the nucleus and that CYFIP2 is capable of undergoing CRM-1/exportin-dependent nucleocytoplasmic shuttling [41]. The roles of both CYFIP and Rac1 in the nucleus are still unclear. Nuclear Rac1 was found in complex with numerous proteins and may thus play different roles [40], including the regulation of cell division [42].

The light membrane fractions contained the largest population of proteins which are linked to the translational machinery, such as ribosomal RPLP0 and the initiation factor eIF5. The high amount of cytoplasmic eIF5 in the absence of FMRP, CYFIP, and ribosomes (including RPLP0) indicates that the association of eIF5 with the translation machinery is independent of FMRP[27].The presence of FMRP, CYFIP, RPLP0, and also eIF5 in the nuclear membrane fraction is most likely based on their association with the rER.

An interesting observation was that nucleolin, another multifunctional RNA-binding phosphoprotein, was found in all FMRP-containing fractions (Fig. 2B). Nucleolin, which has been previously reported to exist in the FMRP-containing protein complexes in murine fibroblasts[26] and human embryonic kidney 293T cells [27], resembles FMRP as it contains an NLS and is able to shuttle between the nucleolus and cytoplasm[43]. It is involved in various processes, including chromatin remodeling, rRNA processing, ribosome biogenesis in the nucleolus, nucleocytoplasmic shuttling of ribosomes, mRNA stabilization, and translation [44]. FMRP contains both a NLS and NES [15] and also undergoes nucleocytoplasmic shuttling [17], [19], [20] and is, like nucleolin [45], associated with mRNP particles and large polyribosomal complexes [11], [12], [13], [14], [46]. Thus, the presence of FMRP in the nucleolar fraction [11] led us to speculate about a possible concerted role of FMRP and nucleolin in nucleocytoplasmic shuttling of ribosomes and escorting mRNAs to the translational machinery. Similar to FMRP, nucleolin is associated with neurodegenerative disorders, such as Huntington's disease, Alzheimer disease, Down syndrome, and progressive supranuclear palsy [47].

Another interesting observation in this study was the association of FMRP with mitochondria (Fig. 1A). To further prove this finding we investigated a possible localization of FMRP in or on mitochondria by isolating highly enriched mitochondria fraction from the heavy membrane fraction of the HeLa cells (Fig. 2A; see Materials and Methods). As illustrated in Figure 2C, a considerable amount of FMRP appeared to exist in the mitochondria like the mitochondrial markers MTCO2 and ACAT1 (Acetyl-CoA acetyltransferase), where also slight amounts of nuclear proteins, such as histone H3 and nucleolin, were also detected. This suggests that the mitochondrial fraction contained nuclear impurity. Nonetheless, our result clearly indicate that FMRP is either physically associated with the outer mitochondrial membrane, eventually as part of p-bodies and stress granules [48], [49] or it is sorted into the mitochondrial matrix. The latter was reported in both EBV-transformed human lymphoblastoid cells using cell fractionation and rat brain neurons using electron microscopy [18]. These observations are in line with the studies suggesting possible mitochondria-associated functions of FMRP, e.g. in preventing apoptosis as a downstream effector of metabotropic glutamate receptors [50] and/or controlling mitochondrial protein translation [50], [51]. Interestingly, partial reduction in the FMRP amount in pre-mutation FMR1 knock-in mice has been shown to correlate with the mitochondrial number and function [52], and to lead to mitochondrial dystrophy [50] and elevated mitochondrial oxidative stress in FXS patients [53]. However, the role of FMRP on/in mitochondria is unclear [51] and requires further investigation.

### FMRP coexists with nucleolin in two distinct, nucleolar and cytosolic complexes

**Figure 3:**
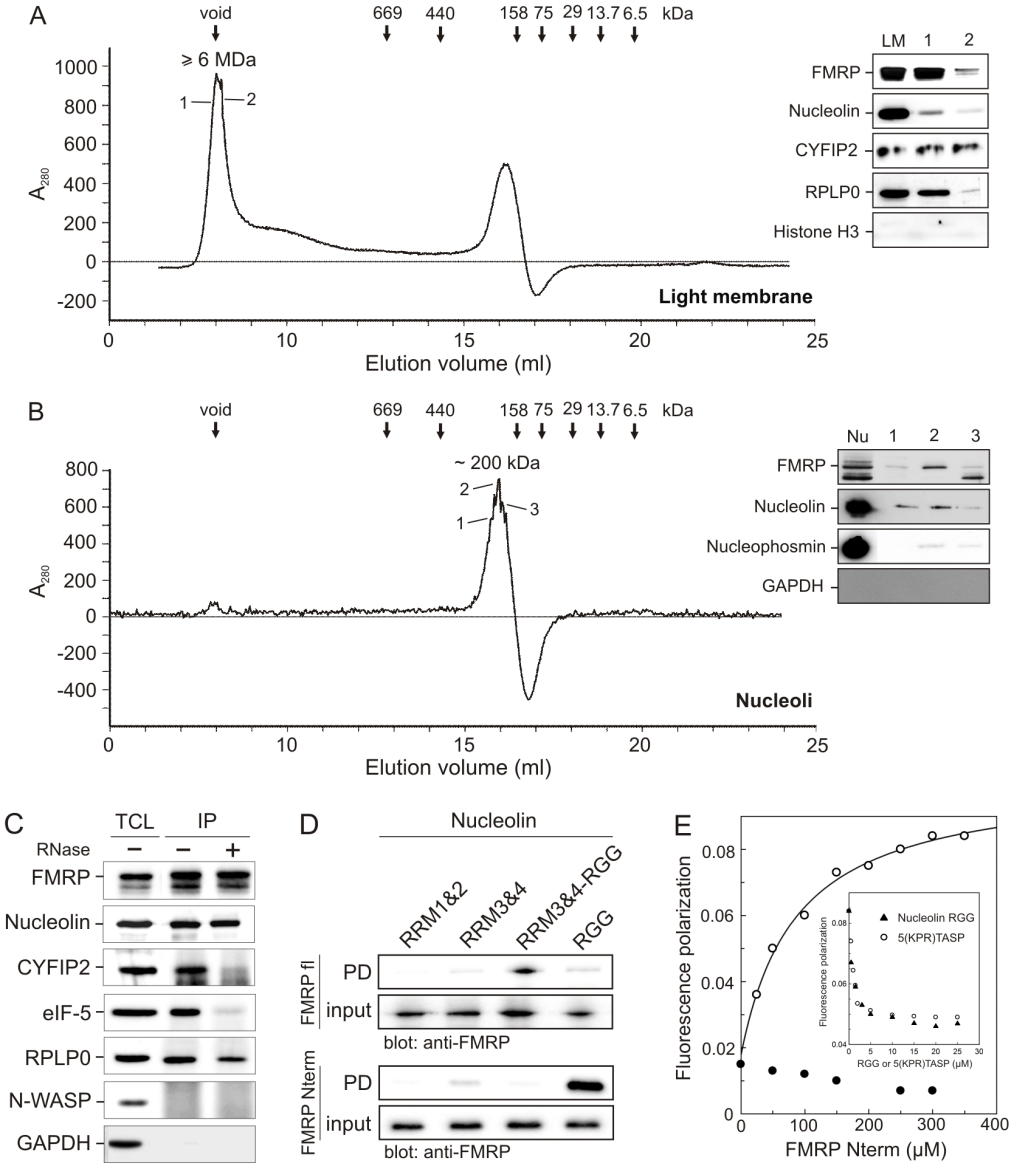
FMRP and nucleolin interact in both cytosolic high molecular weight and nuclear low molecular weight complexes. (A, B) Native FMRP protein complexes were fractionated by loading the light membrane (A) and nucleolar (B) fractions on a superose 6 size exclusion chromatography column. The absorbance of the column eluent at 280 nm (A_280_) was plotted against the elution volume (ml). Different FMRP binding partners and markers are shown, including histone H3 and GAPDH as negative controls in the endomembrane and nucleolar fractions, respectively. The elution positions of standard proteins employed include thyroglobuline (669 kDa), ferritin (440 kDa), aldolase (158 kDa), ovalbumin (75 kDa), carbonic anhydrase (29 kDa), ribonuclease (13.7 kDa), and aprotinin (6.5 kDa). The peak fractions, as indicated by a solid line, were subjected to SDS-PAGE and immunoblotting using antibodies against FMRP (71 kDa), nucleolin (76 kDa), CYFIP2 (146 kDa), RPLP0 (34 kDa) and eIF5 (58 kDa) LM, light membrane; Nu, nucleoli. The molecular mass of the peak fractions is indicated above the peaks. (C) Interaction of FMRP with CYFIP, nucleolin, eIF5, and RPLP0 as analyzed by co-immunoprecipitation. Endogenous FMRP was immunoprecipitated from HeLa cell lysates using an anti-FMRP antibody before and after RNase treatment. FMRP co-precipitated with nucleolin, RPLP0, eIF5, and CYFIP2. Interaction with the latter two proteins was sensitive to RNase treatment. Proteins were visualized by using antibodies against FMRP, nucleolin, eIF5, CYFIP2 and RPLP0. N-WASP and GAPDH were used as a negative IP controls. IP, immunoprecipitation; TCL, total cell lysate. (D) Direct interaction between FMRP and nucleolin. GST pull-down experiments were conducted by mixing bacterial lysate expressing His-tagged FMRP fl (upper panel) or FMRP Nterm (lower panel) with different GST-fused nucleolin proteins (RRM1&2, aa 284–466; RRM3&4, aa 467–644; RRM3&4-RGG, aa 499–710; RGG, aa 645–710) immobilized on GSH sepharose beads. Proteins retained on the beads were resolved by SDS-PAGE and processed for Western blot using a monoclonal antibody against FMRP. Mixed samples before performing pulldown (PD) analysis were used as input control. (E) Low-affinity interaction between the FMRP Nterm and the nucleolin RGG. Fluorescence polarization assay was used as a tool for monitoring the interaction of the FMRP Nterm (increasing concentrations as indicated) with the IAEDANS-labeled fluorescent RGG (0.5 μM) (open circles). As negative controls, FMRP Nterm was titrated into IAEDANS alone (0.5 μM) (closed circles). The inset depicts the displacement of FMRP Nterm from IAEDANS-labeled fluorescent RGG by increasing concentrations of unlabeled RGG and the synthetic peptide construct 5(KPR)TASP.

To further characterize the colocalization and a possible (functional) interaction of FMRP and nucleolin, the light membrane and nucleolar fractions were further analyzed using analytical size exclusion chromatography on a superose 6 HR column calibrated with molecular weight standards (see Material and Methods). As indicated in Figures 3A and 3B, intact FMRP-containing protein complexes of both samples showed a different separation and elution profile. Proteins in the light membrane fraction eluted in the void volume suggesting that the FMRP complexes exhibit a native molecular weight of at least ≥6 MDa (void volume of the superose 6 HR column). Such a cytosolic high molecular weight complex may consist of polyribosomes (∼4.2 MDa/80S ribosome), mRNPs particles and/or additional components or regulators of the translational machinery[11], [13], [35]. In lymphoblasts, FMRP has been shown previously to be part of a protein complex of ≥600 kDa (void volume of the superdex 200 column), which contain the 60 S ribosomal protein RPLP0 as well as the FMRP-related FXR1P and FXR2P [35]. Such a FMRP protein complex has been shown in HeLa cells to be associated with actively translating polyribosomes [13].

In contrast, the FMRP complexes of the nucleolar fraction exhibited a rather low molecular weight of approximately 200 kDa. Immunoblot analysis of both peak fractions further suggested that nucleolin exists in two different FMRP complexes, i.e. in the cytosolic and nucleolar compartments. Based on the detection of nucleolin, CYFIP, and RPLP0 (Fig. 3A) in the light membrane-associated FMRP complex, we assume that this cytosolic high molecular weight complex is even much larger than 5–6 MDa and also contains programmed ribosomes and polysomes [54]. In contrast, the low molecular weight FMRP complex may contain one nucleolin molecule (76 kDa) and two FMRP molecules (71 kDa), as FMRP is able to dimerize *via* its N-terminal domain [15]. This complex may also exist in a 1∶1 stoichiometry since endogenous nucleolin runs approximately at 110 kDa (data not shown).

In order to further characterize the interaction between FMRP and nucleolin as critical translational regulators, we performed immunopreciptation studies using HeLa cell lysates with and without RNase treatment using FMRP-specific antibodies. As indicated in Figure 3C, nucleolin, CYFIP, RPLP0, and also eIF5 were efficiently co-immunoprecipitated and found in a complex with FMRP. Here, CYFIP and eIF5 dissociated almost completely from the complexes when the samples were treated with RNase A. These data strongly suggest that eIF5 and CYFIP association with FMRP within the translation machinery is RNA-dependent. In contrast to the FMRP-eIF5 relationship in translational initiation, which remains unclear, the FMRP-CYFIP complex has been shown to display a translational repression activity [10], which additioanally requires eIF4E [36]. Of particular relevance may be the finding that association of FMRP with nucleolin and RPLP0 was not affected by RNase A treatment (Fig 3C). Consistent with this result, FMRP was previously detected in the same protein complex with RPLP0 [35], as well as with nucleolin, FXR1P, FXR2P, and different mRNAs, including FMRP mRNA [26]. In contrast to the high molacular weight, cytosolic FMRP complex, which most likely controls translation, the role of the low molecular weight nucleolar FMRP-nucleolin complex remains unclear.

In summary our results show that FMRP is predominantly present in two complexes residing in the light membrane and the nucleoli. These macromolecular complexes significantly differ in size and composition. Direct FMRP interaction partners identified by co-immunoprecipitation include nucleolin and RPLP0. In addition, there seems to be an indirect, RNA-mediated interaction of FMRP with CYFIP and eIF5.

### FMRP binds directly to nucleolin

**Figure 4:**
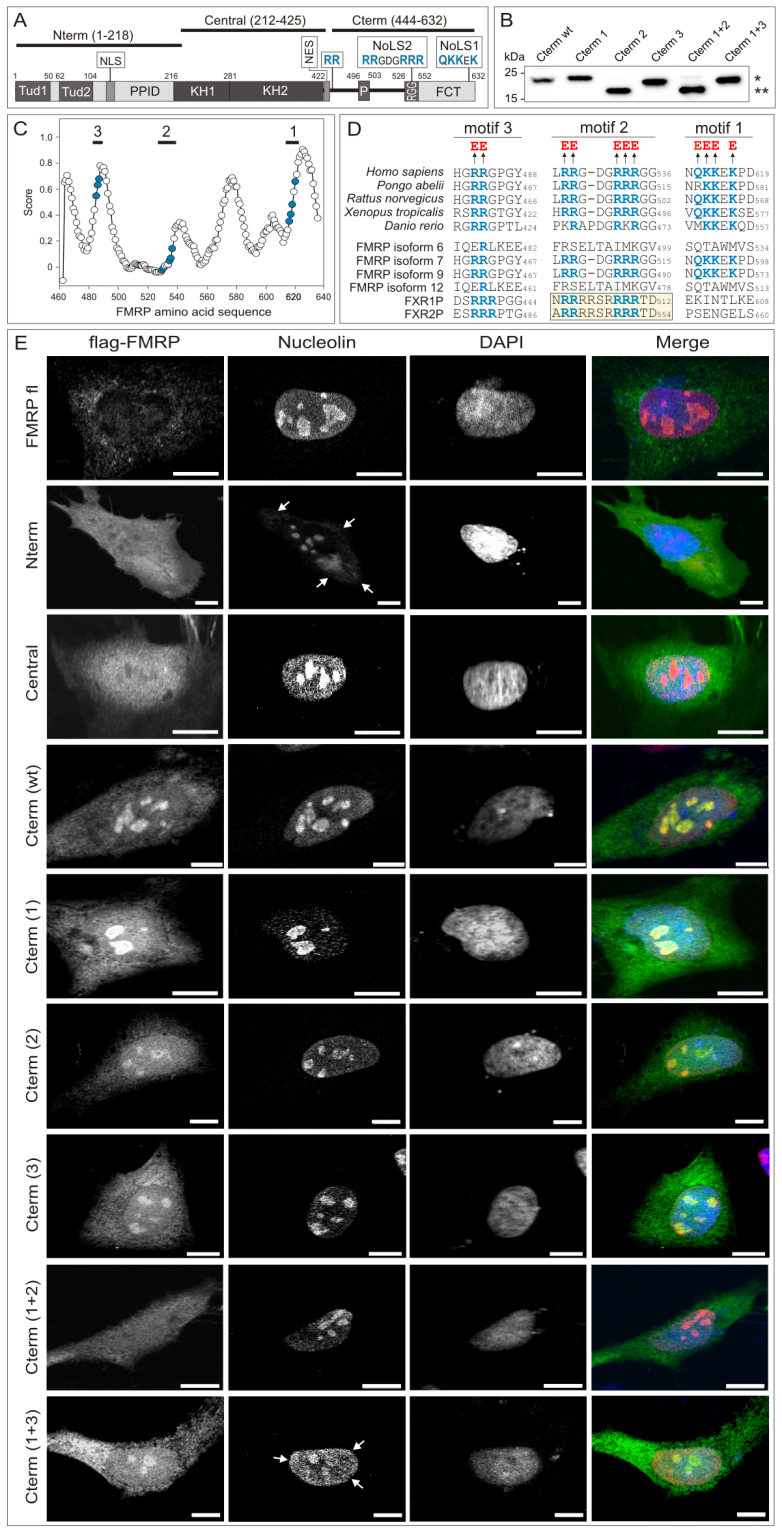
The C-terminal region of FMRP contains evolutionary conserved nucleolar localization signals. (A) Domain organization and motifs of FMRP. Schematic diagram of FMRP architectures highlights major domains and motifs. FCT, FMRP C-terminus; KH1 and KH2, tandem K (described first in the hnRNP K protein) homology domain; NES, nuclear export signal; NLS, nuclear localization signal; NoLS, nucleolar localization signal; PPID, protein-protein interaction domain; RGG, arginine-glycine-glycine region; P, phosphorylation sites; Tud1 and Tud2, tandem Tudor (also called Agenet) domains. The C-terminal region (Cterm; aa 444–632) of FMRP contains two NoLSs, identified in this study. Two further FRMP fragment used were Nterm (1–218) and a Central region (212–425). (B) Overexpression of the Cterm wild-type (wt) and its variants on HeLa cells. Cterm 1: QKKEK changed to EEEeE; Cterm 2: RRGDGRRR changed to EEgdgEEE; Cterm 3: RR changed to EE; Cterm 1+2: a combination of Cterm 1 and 2 mutations; Cterm 1+3: a combination of Cterm 1 and 3 mutations. Cterm 2 and Cterm 1+2 revealed a change in protein mobility (**) as compared to the wild-type and the other variants (*). (C) NoLS prediction of FMRP Cterm using the NoLS predictor program [58]. Graph shows the probability of NoLS distribution (represented by score) plotted against the amino acid sequence of FMRP Cterm (444–632). Three motifs and critical positively charged residues are marked blue. (D) Multiple sequence alignment of the three predicted NoLS motifs 1, 2, and 3 of FMRP Cterm from different species (upper panel) as well as FMRP transcripts and homologous proteins (lower panel). Basic residues (blue), which are changed to glutamic acids (red) are highlighted. Upper panel: FMRP sequences from different species are human (accession number 544328), orangutan (197102198), rat (30794228), frog (53749722) and zebrafish (23308667). Lower panel: FMRP transcripts and homologous proteins are transcript 6 (297374777), 7 (297374779), 9 (297374791) and 12 (297374789) as well as FXR1P (61835148) and FXR2P (259013556). (E) Nucleolar localization of FMRP. cLSM images of HeLa cells transfected with FMRP fl, Nterm, Central, Cterm (wt) and Cterm variants (anti-flag; green channel) costained with endogenous nucleolin (anti-nucleolin; red channel) and DNA (DAPI; blue channel) revealed that Cterm (wt), (1), (2), (3) and (1+3) colocalize with nucleolin in the nucleolus. In contrast this colocalization was absent in the case of Cterm (1+2). Cytoplasmic distribution of FMRP Nterm and the subnuclear distribution of endogenous nucleolin are highlighted by arrows. Scale bar: 10 μm.

Our data about the existence of different FMRP–nucleolin protein complexes prompted us to map the FMRP-nucleolin interaction at the protein level. We performed GST pulldown experiments under cell-free conditions by using His_6_-tagged full length (fl) as well as the N-terminal region (Nterm) of FMRP (aa 1–218; Fig. 4A) and various nucleolin subdomains as Gluthation-S-transferase (GST) fusion proteins. As indicated in Figure 3D, both FMRP variants were able to directly bind to nucleolin subdomins, although with different patterns. FMRP fl strongly bound a construct encompassing the RRM3&4-RGG (aa 499–710) domains of nucleolin and to a weaker extent also to RRM3&4 (aa 467–644) domains and the C-terminal RGG domain (aa 645–710) of nucleolin. This picture was, however, greatly different for interaction studies using the N-terminus of FMRP. The strongest interaction was found between the N-terminus of FMRP and the RGG domain of nucleolin. Binding of the FMRP N-terminus to the RRM3&4 fragment was as weak as that of FMRP fl, and binding of RRM3&4-RGG domains was also not much pronounced. Both FMRP constructs did not reveal any interaction with RRM1&2 (aa 284–466) domains of nucleolin (Fig. 3D).

In order to determine the binding affinity between FMRP Nterm and nucleolin RGG, we used the advantage of fluorescence polarization. Therefore, we labeled RGG with the fluorescence reporter group IAEDANS. As shown in Figure 3E, we notably observed an incremental increase in fluorescence polarization in the presence of increasing amounts of FMRP Nterm but not with IAEDANS alone. From this data a dissociation constant (K_d_) value of 87 μM was calculated illustrating a low-affinity FMRP-nucleolin interaction. Competition experiments were next performed to prove this complex formation using the purified, unlabeled RGG domain of nucleolin and a synthetic peptide construct 5(KPR)TASP, which has been reported previously to bind nucleolin [55]. As indicated in Figure 3E, the unlabeled RGG domain and the peptide construct efficiently displaced FMRP Nterm from its complex with the fluorescent RGG domain by binding to FMRP Nterm and the fluorescent RGG domain, respectively.

The nucleolin-binding N-terminus of FMRP harbors due to its various subdomains different interaction characteristics (Fig. 4A) [10]. It contains two conserved Tudor domains (Tud1 and Tud2; also called N-terminal domain of FMRP 1 and 2 or NDF1 and NDF2 [16]) that are part of the proposed royal family of protein domains, also including Agenet, MBT, PWWP, and chromo domains [56]. The Tudor domains of FMRP, FXR1P, and FXR2P have been shown to selectively bind trimethylated lysines peptides derived from histones H3K9 and H4K20 [16], [57]. The Tud2 domain of FMRP has also been shown to physically bind 82-FIP (82-kDa FMRP Interacting Protein) [16], which has been identified as a component of FMRP-containing mRNP complexes [25]. The Tud2 domain of FMRP has been proposed to be a stronger target for interactions as compared to Tud1, possibly because of its plasticity and availability of exposed hydrophobic cavities [16].

Taken together, our findings suggest that the N-terminus of FMRP is responsible for its physical interaction with nucleolin. The FMRP binding epitope on nucleolin could be identified as the RGG region, which has been implicated in facilitating a variety of protein and RNA interactions [44].

### FMRP contains a functional nucleolar localization signal (NoLS)

The fact that FMRP was clearly detectable in the nucleolar fraction similar to nucleolin (Fig. 2B), and that both proteins directly interact with each other (Fig. 3D), was remarkable, especially because FMRP was found to be associated with the granular component of the nucleolus using immunogold labeling and electron microscopy [21]. In order to clarify the significance of these observations, we subjected FMRP to a mutational analysis by studying the colocalization of different FMRP subdomains with endogenous nucleolin in HeLa cells. Most remarkably, we found that in contrast to FMRP full-length (fl), the N-terminus (called Nterm; aa 1–218), and the central KH domains (called central; aa 212–425), a C-terminal region of FMRP (called Cterm; aa 444–632) clearly colocalized with nucleolin in the nucleolus (Fig. 4E), when overexpressed in HeLa cells. These data strongly suggest that FMRP Cterm must encompass one or more functional NoLS motifs.

Therefore, we next screened for the presence of NoLS motifs in FMRP Cterm by employing a NoLS prediction software (http://www.compbio.dundee.ac.uk/www-nod) [58]. This analysis showed that there are at least three putative NoLS motifs at the C-terminus of FMRP (Fig. 4C), which are partially conserved in other organisms and within different FMRP isoforms and related proteins. One of these NoLS motifs (motif 2, Fig. 4D) has been previously described for the FMRP-related proteins FRX1P and FRX2P [59]. Interestingly, FMRP isoforms 6 and 12, which lack both NoLS1 and NoLS2 (Fig. 4D), have been very recently reported to localize to Cajal bodies in the nucleus [60], due to a different C-terminal region. Cajal bodies participate in the biogenesis of small nuclear ribonucleoproteins (snRNPs) with which FMRP isoforms are associated [61].

To analyze the potential roles of these motifs, we substituted the positively charged residues in all three motifs with negatively charged glutamic acids (E) in the context of the Cterm construct (Fig. 4D). Transient transfection experiments of HeLa cells have shown that Cterm mutants of motifs 1 and 2 (Cterm 1 and 2), but not motif 3 (Cterm 3), resulted in a significant reduction of their nucleolar localization as compared to the wild-type situation (Cterm wt) (Fig. 4E). This effect was stronger when we combined the substitution of both motifs 1 and 2. Consequently, overexpressed Cterm 1+2, when compared to Cterm 1+3, was localized at other subcellular regions, but not in the nucleolus (Fig. 4E). In addition, a subnuclear distribution of nucleolin was observed when Cterm 1+3 was overexpressed (Fig. 4E). Thus, our data clearly demonstrate the existence of at least two evolutionary conserved and functional motifs (NoLS1 and NoLS2) that are crucial for nucleolar localization of FMRP.

The fact that FMRP, which contains functional NLS and NoLSs, was predominantly found in the cytoplasm (Figures. 1A, 1B and 4E) can be explained by the presence of different nuclear export mechanisms [17], [19], [20]. However, it is rather intriguing that nucleolin, which lacks NES and NoLS (data not shown), almost completely localized to the nucleolus (Figs.1A, 1B and 4E). The presence of two NoLS motifs in FMRP and the fact that it selectively binds nucleolin led us to speculate that FMRP may facilitate nuclear and nucleolar import of nucleolin in a kind of piggyback mechanism. Next to the Tudor domains is a non-canonical NLS within the FMRP Nterm [17], [19], [62], which most likely facilitates FMRP shuttling into the nucleus [10], [37]. This piggypack hypothesis is nicely supported by the observation that overexpression of FMRP Nterm resulted in the subcellular redistribution of nucleolin (Fig. 4E). FMRP Nterm competes for nucleolin binding with endogenous FMRP and prevents the FMRP-mediated nucleolin targeting to the nucleolus. This function of FMRP obviously is rather significant especially because a missense mutation in the NLS of the FMR1 gene, altering a conserved arginine residue at position 138 to glutamine (R138Q), may represent an important cause associated with developmental delay [63]. An rRNA-mediated nucleolar localization of nucleolin has been proposed in an early study that has shown that the C-terminal regions of nucleolin, containing the RRMs and the RGG domain, are essential for nucleolar accumulation of nucleolin [64]. Thus, it is very likely that a concerted interaction of FMRP with nucleolin regulates the transport of rRNAs and ribosomal proteins towards the nucleolus and the export of ribosomes from the nucleolus as well as mRNAs and mRNP particles from the nucleus [10], [26], [44], [65]. It is important to note that nucleolin may be addressed in the nucleolus by a FMRP-independent mechanism since it has its own NLS [64] Accordingly, nucleolin and FMRP could assemble in the nucleoplasm or in the nucleolus in order to accomplish ribosomal biogenesis and ribosome or mRNP particle export.

An important question to be addressed is why FMRP is predominantly cytoplasmic, although it contains functional NES and NoLS motifs. Several explanations for this paradox are proposed: (1) The NLS and eventually the NoLS motifs of FMRP may be masked in the context of FMRP fl through an intramolecular or intermolecular mechanism that need to be released for example by posttranslational modifications [19]. FMRP phosphorylation of conserved residues (Ser497, Ser500, Thr502 and Ser504) is located within the phosphorylation (P) region close to the identified NoLS motifs (Fig. 4A) [46], [66] and may also have modulatory impacts on the FMRP redistribution [67]. (2) Interestingly, NoLS2 is an integral part of the RGG region of FMRP, and arginine methylations in and around this region have been reported to regulate the association of FMRP with polyribosomes and mRNA [48], [54]. This may suggest that nucleolar localization of FMRP, possibly in complex with nucleolin, may well be a prerequisite for FMRP association with and nuclear export of target mRNAs and ribosomes [17], [68]. (3) The fact that FMRP fl predominantly localizes in the cytosolic compartments supports the notion that FMRP, due to its two NES motifs, underlies an efficient mechanism for nuclear export. Consistent with this, deletion of the NES motifs has been shown to result in FMRP accumulation in the nucleus [17].

## Conclusions

The discovery of the FMR1 gene defects over twenty-two years ago [69] has led to significant advances in understanding the critical role of FMRP in synaptic plasticity and the molecular events of the fragile X mental retardation syndrome [2], [6], [14], [67]. Despite the bulk of data concerning the molecular properties of FMRP and despite the growing body of evidence on its functional significance especially in neurons, the molecular mechanisms by which FMRP plays a role in RNA transport and metabolism, translation regulation, cytoskeleton remodeling, and cell motility still remain to be elucidated.

This study describes the thorough investigation of physical and functional niches of FMRP by analyzing the subcellular distribution of endogenous FMRP and its complexes under native conditions in HeLa cells. Confocal microscopy imaging subcellular fractionation and precipitation experiments provide valuable insights into (i) FMRP association with various nuclear and cytosolic fractions of variable molecular weights, (ii) uncovered a direct interaction between FMRP N-terminus and the RGG domain of nucleolin, and (iii) identified the existence of two functional NoLSs at the C-terminus of FMRP. Our data open new perspectives of a possible mechanistic link between nucleolar ribosome biogenesis, RNA shuttling and the cytoplasmic translational machinery that may be dependent on distinct functional subsets of FMRP-nucleolin complexes.

This study also demonstrates the presence of FMRP-containing complexes in the nucleus and the cytoplasm. These complexes contain nucleolin and other crucial factors for RNA processing and translational control. A direct interaction of FMRP with nucleolin was identified by RNase digestion experiments and interaction studies using purified proteins. We were further able to identify the responsible binding epitopes as the N-terminus of FMRP and the RGG domain of nucleolin. A potential functional role the FMRP-nucleolin complex formation may be nucleocytoplasmic shuttling of nucleolin provided by the presence of functional NLS, NoLSs and NESs existing in FMRP.

Future biophysical investigations of FMRP beyond differential cell fractionation and size exclusion chromatography will eventually require the use of blue native polyacrylamide gel electrophoresis, which is in combination with mass spectroscopy a powerful strategy for further separation and identification of native multiprotein FMRP complexes. These will provide an essential framework for uncovering the molecular niches and the physical environment of FMRP endowed of specific, molecular properties, and may ultimately open new perspectives in elucidating the molecular mechanisms of FMRP regulation and function.

## Materials and Methods

### Constructs

Nterm (aa 1–218) and Cterm (aa 444–632) of human FMRP, RRM3&4-RGG (aa 499–710) and RGG (aa 645–710) of human nucleolin were amplified by standard PCR and cloned into pGEX-4T1 and pGEX-4T1-Ntev. Full length FMRP (FMRP-fl; aa 1–632) was cloned into pET23b as a His-tag fused protein. Moreover, FMRP-fl, Nterm (aa 1–218), Central (aa 212–425) and Cterm (aa 444–632) were cloned into pcDNA 3.1-FLAG. RRM1&2 (aa 284–466), RRM3&4 (aa 467–644), RRM3&4-RGG (aa 499–710) and RGG (aa 645–707) of human nucleolin were kindly provided by F. Carrier [70].

### Cell culture

Various cell lines, including Cos-7, HEK 293, HeLa and NIH3T3, were obtained from the German Collection of Microorganisms and Cell Cultures (DSMZ, Braunschweig, Germany), MDCK II from the American Type Culture Collection (ATCC, Manassas, USA), and wild-type murine embryonic fibroblasts (MEFs) from our laboratory. All cell lines were grown in DMEM supplemented with 10% fetal bovine serum (FBS) (Life Technologies, Darmstadt, Germany) and penicillin/streptomycin as antibiotics. Trypsin/EDTA was from Genaxxon Bioscience GmbH, Ulm, Germany.

### Antibodies and fluorescent probes

Anti-FMRP (F6072) was purchased from US Biological (Swampscott, United States); anti-FMRP (ab17722), anti-FMRP (phospho S499) (ab48127), anti-calreticulin (ab4), anti-Lamin B1 (ab16048), anti-RPLP0 (ab88872), anti-CYFIP2 (AB95969), anti-nucleolin (ab22758), anti-MTC02 (AB3298), anti-nucleophosmin (ab10530), anti-ACAT1 (ab71407) and anti-EEA1 (ab2900) were purchased from Abcam (Cambridge, United Kingdom); anti-eIF5 (SC-28309), anti-N-WASP (sc-100964), and anti-Gα_q/11_ (sc-392) were from Santa Cruz biotechnology (Texas, USA); anti-nucleoporin p62 (610498) and anti-Rac1 (#610651) were from BD Transduction (New Jersey, USA); anti-Histone H3 (*#* 9715) and anti-GAPDH (*#* 2118) were from Cell Signaling (Boston, USA) and anti-Actin (*#* MAB1501) from Millipore (Temecula, U.S.A). Anti-Na^+^/K^+^ ATPase (A276), anti-FLAG (F3165) and DAPI were obtained from Sigma-Aldrich. Alexa fluor 546 phalloidin and the secondary antibodies Alexa fluor 488 goat anti-rabbit IgG and Alexa fluor 633 goat anti-mouse IgG were obtained from Molecular Probes (Oregon, USA).

### Proteins

Escherichia coli BL21(DE3) pLysS, BL21(DE3) CodonPlus-RIL, or BL21(Rosetta) strains transformed with the respective construct were grown until an OD600 value of 0.7 and thereafter induced with 0.1 mM isopropyl-β-D-thiogalactopyranoside (IPTG) overnight at 25°C. All proteins were purified as described as described [71], [72].

### Transient transfection

HeLa cells were transfected using the TurboFect (Thermo Scientific) transfection reagent according to the manufacturer's instructions in 6-well plates employing 4 μg DNA per transfection.

### Immunofluorescence microscopy

HeLa cells grown on glass coverslips were fixed with 4% paraformaldehyde for 20 min, permeabilized with 0.25% triton-X100 in PBS for 10 min, and thereafter blocked for 1 h in a solution containing 3% BSA in 0.25% triton-X100/PBS. Cells were incubated with primary and secondary antibodies for 1 h and finally counterstained with DAPI (4′,6-diamidino-2- phenylindole dihydrochloride) for 5 min and mounted using the prolong gold anti-fade reagent (Molecular Probes, Eugene, USA). Images were obtained as single optical slides using a LSM510-Meta confocal microscope (Zeiss, Jena, Germany) equipped with a 40x/1.3 immersion objective and excitation wavelengths of 364 nm, 488 nm, and 546 nm.

### Subcellular fractionation by differential centrifugation

A differential centrifugation method was combined with the use of sucrose cushions in this study to fractionate HeLa cells. In addition, we avoided detergents and sonication in order to keep subcellular protein complexes intact. HeLa cells were homogenized by using a pre-chilled 7 ml Dounce homogenizer in a detergent-free lysis buffer containing 10 mM Tris/HCl (pH 7.4), 10 mM NaCl, 0.5 mM MgCl_2_, and EDTA-free protease inhibitor cocktail (Roche, Berlin, Germany). The homogenates were centrifuged at 2,000xg for 5 min at 4 °C. The pellets were resuspended in 250 mM sucrose solution containing 10 mM MgCl_2_ and centrifuged through an 880 mM sucrose cushion containing 0.5 mM MgCl_2_ at 1,200xg for 10 min in order to obtain the crude nuclear and cytoplasmic fractions. The supernatants were further subjected to a 16,000xg centrifugation step for 10 min to isolate the heavy membrane pellet and the post-nuclear supernatant. The post-nuclear supernatants were then centrifuged for 1.5 h at 130,000×g. The resulting pellets contained the light membrane fraction and polysomes. Nuclei were resuspended in lysis buffer and gently homogenized using a Balch homogenizer (clearance of 8 μm) and 8-10 up-and-down strokes. Homogenized nuclei were centrifuged through a cushion of 880 mM sucrose containing 0.5 mM MgCl_2_ at 2000×g for 20 min to isolate the nucleolar pellet and post-nucleolar supernatant. The post-nucleolar supernatant was finally centrifuged for 1.5 h at 130,000×g. The resulting pellets contained the nuclear membranes and the supernatants the nucleoplasmic fractions. All fractionation steps were carried out at 4°C. Protein concentration of all fractions was determined by the Bradford assay (Bio-Rad, Hercules, CA). All fractions were divided into two samples. One sample was mixed with 5× SDS-PAGE loading buffer and 6 μg of total protein was subjected to SDS-PAGE. The other sample was analyzed using size exclusion chromatography.

### Isolation of mitochondria

HeLa cell mitochondria were isolated from the heavy membrane fraction (see above) using a modified protocol described previously [73]. This fraction was subjected to PEB buffer (PBS buffer, pH 7.2, 2 mM EDTA and 0.5% bovine serum albumin) and incubated with 30 μl anti-TOM22 MicroBeads (Miltenyi Biotec, Bergisch Gladbach, Germany) for 1 h at 4 °C. The mixture was loaded onto a pre-equilibrated MACS column (Miltenyi Biotec), which was placed in the magnetic field of a MACS separator (Miltenyi Biotec). The column was washed three times with 3 ml PEB buffer and retained mitochondria were finally eluted in a volume of 1.5 ml. Mitochondrial solution was centrifuged at 13,000xg for 1 min. The mitochondria-containing pellet was washed two times using 0.32 M sucrose, 1 mM EDTA, and 10 mM Tris/HCl. After centrifugation (13,000xg, 1 min) the mitochondrial pellet was resuspended in 20 mM Tris/HCl (pH 7.5) and equal protein amount of mitochondrial fraction and total cell lysate were subjected to SDS-PAGE and immunoblotting analysis.

### Analytical size exclusion chromatography (aSEC)

Analytical size exclusion chromatography was employed for further separation of FMRP complexes in the endomembrane and nucleolar fractions using a superose-6 HR 10/30 column (GE Healthcare, Uppsala, Sweden) and a buffer containing 30 mM HEPES (pH 7.6), 5 mM MgCl_2_, 150 mM NaCl, and 3 mM DTT. The optimal separation range of the column is 5 kDa to 5 MDa, with an exclusion limit of 40 MDa (GE Healthcare, Uppsala, Sweden).The flow rate was maintained at 0.5 ml/min. Fractions were collected at a volume of 0.5 ml. Peak fractions were visualized by 15% SDS-PAGE gel and staining using Coomassie brilliant blue (CBB).

### Immunoprecipitation

For immunoprecipitation of FMRP protein complexes from cellular extracts, HeLa cells were lysed in a buffer containing 20 mM Tris/HCl (pH 7.5), 150 mM NaCl, 1 mM EDTA, 1% triton X-100, 2.5 mM Na-pyrophosphate, 1 mM β-glycerophosphate, 1 mM sodium vanadate, one EDTA-free protease inhibitor cocktail tablet (Roche, Mannheim, Germany), and 70 U RNase A (Qiagen, Hilden, Germany) in order to determine RNA dependent interacting partners of FMRP. Lysates were centrifuged at 10,000xg for 10 min. The supernatant was precleared with protein A/G plus-agarose (sc-2003, Santa Cruz Biotechnology, Texas, USA) and then incubated with an anti-FMRP antibody (5 μg/ml; ab17722; Abcam, Cambridge, UK) overnight at 4 °C. Thereafter, protein A/G plus-agarose beads were added to the lysate for 1 h before recovering the beads by centrifugation at 664xg for 5 min at 4°C. The beads were washed 4-times in the lysis buffer, resuspended in SDS-PAGE loading buffer and analyzed by SDS-PAGE and Western blotting using a BioRad Mini-PROTEAN system (BioRad, Hercules, CA).

### Pull-down assay

GST pull-down experiments were conducted by adding 500 μg of bacterial lysate expressing His-tagged FMRP fl or 50 μg FMRP Nterm purified protein with 25 μg of different GST-fused nucleolin proteins (RRM1&2, aa 284–466; RRM3&4, aa 467–644; RRM3&4-RGG, aa 499–710; RGG, aa 645–710) immobilized on 30 μl glutathione-conjugated Sepharose 4B beads (Macherey-Nagel, Duren, Germany). The mixture was incubated at 4°C for 45 min in buffer, containing 50 mM Tris/HCl, pH 8.0, 150 mM NaCl, 1 mM DTT, 5% glycerol. After washing for five times with the same buffer proteins retained on the beads were resolved by SDS-PAGE and processed for immunoblotting using a monoclonal antibody against FMRP. Mixed samples prior to pull-down (PD) analysis were used as input controls.

### Peptide synthesis

The template assembled synthetic peptide 5(KPR)TASP [74], [75] was synthesized by manual solid-phase peptide synthesis on Rink Amide resin (Novabiochem, 100–200 mesh, 0.59 mmol/g loading) using standard Fmoc/HBTU peptide coupling conditions. Briefly, the resin (200 μmole) was pre-swollen by suspending in 3 ml of *N*-methyl-2-pyrrolidone (NMP) for 10 min followed by deprotection of the fluorenylmethyloxycarbonyl (Fmoc)-protecting group using 3 ml of 20% piperidine (v/v) in NMP (2×5 min). Each amino acid coupling was performed by mixing 2 ml of a 0.4 M stock solution of *O*-Benzotriazole-*N, N, N′, N′*-tetramethyluronium-hexafluoro-phosphate (HBTU) with 4 ml of a 0.2 M NMP stock solution of the amino acid, followed by 2 ml of a 1.6 M NMP stock solution of *N,N*-diisopropylethylamine (DIPEA). The reaction mixture was added immediately to the resin and the reaction vessel agitated at ambient temperature for 30 min. For the synthesis of the peptide backbone, the *N*-α-Fmoc-protected amino acid building blocks were introduced sequentially and in the following order (single coupling): Fmoc-Cys(Trt)-OH, Fmoc-Gly-OH, Fmoc-Lys(Alloc)-OH, Fmoc-Glu(*t*Bu)-OH, Fmoc-Pro-OH, Fmoc-Gly-OH, Fmoc-Lys(Alloc)-OH, Fmoc-Lys(Boc)-OH, Fmoc-Lys(Alloc)-OH. Prior to the introduction of the peptide side-chains, orthogonal cleavage of the allyloxycarbonyl (alloc)-protecting groups was performed by suspending the pre-swollen resin (10 min, dichloromethane, CH_2_Cl_2_) in a CH_2_Cl_2_ solution of *tetrakis-*(triphenylphosphine)palladium(0) (0.1 eq. per alloc group) and *N,N′*-dimethylbarbituric acid (5 eq. per alloc group), according to a recently described protocol [76]. For the synthesis of the peptide side-chains, triple couplings were necessary to introduce the Fmoc-R(Pbf)-OH, Fmoc-Pro-OH and Fmoc-Lys(tBu)-OH building blocks. The 5(KPR)TASP peptide was cleaved from the resin using a 92.5/2.5/2.5/2.5 (v/v) mixture of trifluoroacetic acid (TFA)/H_2_O/triisopropylsilane (TIS)/ethanedithiol (EDT), and then precipitated in ice-cold diethyl ether. The peptide construct was then purified by reverse-phase HPLC using an Alltima HP C18 column (5 μm, length 125 mm, ID: 20 mm) and 0.1% TFA in H_2_O/MeCN as mobile phase. The pure peptides were analyzed by LC-MS using a Shimadzu LC Controller V2.0, LCQ Deca XP Mass Spectrometer V2.0, Alltima C18-column 125×2.0 mm, Surveyor AS and PDA with solvent eluent conditions: CH_3_CN/H_2_O/1% TFA (C_132_H_246_N_51_O_27_S_1_, [M+3H]^3+^ Calculated: 1003.31; Measured: 1003.80). The identity of the peptides was verified by MALDI (C_132_H_244_N_51_O_27_S_1_, [M+H]^+^ Calculated: 3008.98; Measured: 3007.98). The Rink Amide resin and all amino acid building blocks were purchased from Novabiochem®. HBTU, *N*,*N*-diisopropylamine, *N*-methyl-2-pyrrolidone, CH_2_Cl_2_, HPLC-grade CH_3_CN and HPLC-grade TFA were all purchased from Biosolve B.V. Diethyl ether was purchased from Actu-All Chemicals. [(PPh_3_)_4_Pd], *N,N*′-dimethlybarbituric acid, ethanedithiol, and triisopropylsilane were all purchased from Sigma-Aldrich. H_2_O refers to Millipore grade distilled water.

### Fluorescence polarization

Fluorescence labeling of the nucleolin RGG with the fluorescence reporter group N-(Iodoacetaminoethyl)-1-naphthylamine-5-sulfonic acid (IAEDANS; Sigma, Deisenhofen, Germany) was performed as previously described [72]. Increasing amounts of FMRP Nterm (50, 100, 150, 200, 250, 300 and 350 μM) were titrated into IAEDANS-labeled fluorescent RGG (0.5 μM) in a buffer, containing 30 mM Tris/HCl (pH 7.5),150 mM NaCl, 5 mM MgCl_2_, 1 mM tris-(2-carboxyethyl) phosphine and a total volume of 200 μl at 25 °C using a Fluoromax 4 fluorimeter. The concentration dependent binding curve was fitted using a quadratic ligand binding equation.
